# Medical Professional Reports and Child Welfare System Infant Investigations: An Analysis of National Child Abuse and Neglect Data System Data

**DOI:** 10.1089/heq.2023.0136

**Published:** 2023-09-29

**Authors:** Frank Edwards, Sarah C.M. Roberts, Kathleen S. Kenny, Mical Raz, Matty Lichtenstein, Mishka Terplan

**Affiliations:** ^1^ Rutgers University–Newark, Newark, New Jersey, USA.; ^2^ Advancing New Standards in Reproductive Health (ANSIRH), Department of Obstetrics, Gynecology, and Reproductive Sciences, University of California San Francisco, Oakland, California, USA.; ^3^ University of Manitoba, Winnipeg, Canada.; ^4^ University of Rochester, Rochester, New York, USA.; ^5^ Florida Atlantic University, Boca Raton, Florida, USA.; ^6^ Friends Research Institute, Baltimore, Maryland, USA.

**Keywords:** child welfare, medical professionalism, pregnancy, racial inequity, substance use

## Abstract

**Background:**

Medical professionals are key components of child maltreatment surveillance. Updated estimates of reporting rates by medical professionals are needed.

**Methods:**

We use the National Child Abuse and Neglect Data System (2000–2019) to estimate rates of child welfare investigations of infants stemming from medical professional reporting to child welfare agencies. We adjust for missing data and join records to population data to compute race/ethnicity-specific rates of infant exposure to child welfare investigations at the state-year level, including sub-analyses related to pregnant/parenting people's substance use.

**Results:**

Between 2010 and 2019, child welfare investigated 2.8 million infants; ∼26% (*n*=731,705) stemmed from medical professionals' reports. Population-adjusted rates of these investigations stemming doubled between 2010 and 2019 (13.1–27.1 per 1000 infants). Rates of investigations stemming from medical professionals' reports increased faster than did rates for other mandated reporters, such as teachers and police, whose reporting remained relatively stable. In 2019, child welfare investigated ∼1 in 18 Black (5.4%), 1 in 31 Indigenous (3.2%), and 1 in 41 White infants (2.5%) following medical professionals' reports. Relative increases were similar across racial groups, but absolute increases differed, with 1.3% more of White, 1.7% of Indigenous, and 3.1% of Black infants investigated in 2019 than 2010. Investigations related to substance use comprised ∼35% of these investigations; in some states, this was almost 80%.

**Discussion:**

Rates of child welfare investigations of infants stemming from medical professional reports have increased dramatically over the past decade with persistent and notable racial inequities in these investigations.

## Background

The US child welfare system is composed of state and local agencies that receive reports of alleged child maltreatment, dispatch social workers to investigate allegations, make decisions to place children into foster care, and monitor families while under investigation. About 3.5 million children are subjects of child maltreatment investigations and ∼600,000 children are in foster care annually.^1,2^ American Indian and Alaska Native (AIAN), and Black children are separated from their families far more often than White children, and child welfare systems have disproportionately impacted these communities.^3–9^

Research has identified negative consequences of child maltreatment investigations on maternal and child health.^4,10–21^ Research also consistently finds that fear of being reported to child welfare is a significant barrier to prenatal care for some pregnant people, particularly those who use drugs and alcohol, and is a reason people physically avoid and emotionally disengage from care.^17,18,22–25^ Furthermore, a growing body of research documents how child welfare involvement can harm rather than improve children's health.^26–32^ Because of these and other adverse consequences from reporting, the American College of Obstetricians and Gynecologists has argued against linking pregnancy and postpartum care to punitive and criminalizing surveillance policies, including the routine child welfare investigation for substance use.^24,33^

Despite these medical professional association concerns, medical professionals have been a cornerstone of child maltreatment surveillance.^1,34^ Social scientists have identified differential surveillance by medical personnel as one source of inequitable child welfare involvement and outcomes.^16,17,22,34,35^ Specifically, the racially uneven application of infant and/or prenatal drug testing in medical settings is one pathway through which medical professionals contribute to inequities in surveillance and then subsequent child welfare investigation and separation of families.^36,37^

Over the past 20 years in the United States, federal and state policies related to child welfare reporting and investigation of infants have changed dramatically following the reauthorization of the Child Abuse Prevention and Treatment Act (2010) and the Comprehensive Addiction and Recovery Act (2016). In 2000, 12 states had policies mandating health care providers to report birthing people related to substance use for child welfare purposes. By 2020, 27 states and the District of Columbia (D.C.) mandated such reporting.^38–40^ Although policies related to reporting substance use during pregnancy have increased and appear to contribute to increases in reporting,^41^ we have little information about the: numbers of infants reported, extent to which racial inequities in reporting exist, and variations in reporting over time and across states. Earlier research has documented that child welfare reports owing to a birthing person's substance use range from 0.3% to 1.6% of live births.^42,43^ There are significant racial inequities in this reporting. Some studies have found that Black infants are more likely to be screened for substance exposure,^44,45^ and to be reported to child welfare agencies for prenatal substance exposure.^36^ Other studies have found no difference by race in reporting or infant removal.^43,46^ Most evidence, though, comes from single states or single counties and were collected before the increase in state policies mandating reporting.

This article uses national administrative data to assess prevalence of child welfare investigations of infants resulting from reports by medical professionals and racial inequities in these investigations across states and over time. We also examine overall and racial inequities in rates of investigations of infants related to prenatal substance exposure.

## Methods

Data come from the National Child Abuse and Neglect Data System (NCANDS).^47^ NCANDS is an annual census of investigations of alleged child maltreatment by state and county child welfare agencies. Agency case workers collect case-level and child-level information on investigated children and families, then states report these data annually to the U.S. Children's Bureau. NCANDS provides the most comprehensive national data on the >4 million child subjects of maltreatment investigations annually in the United States. NCANDS does not record detailed information on “screened-out” cases, that is, cases that do not result in child welfare investigations, at least 2 million annually.^1^

**FIG. 1.**
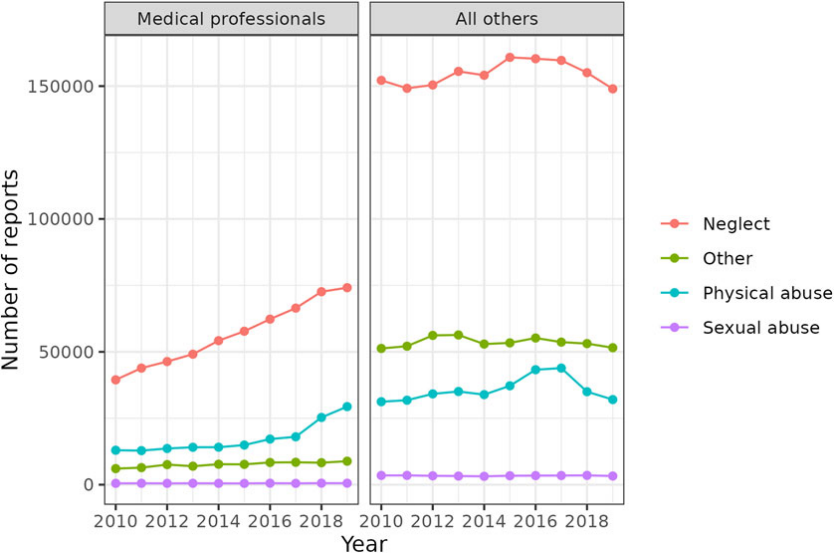
Count of U.S. child welfare investigations of infants (age less than 1 year) 2010–2019 by alleged maltreatment type (a single investigation may allege multiple types of maltreatment). *Left panel*: investigations initiated by a medical professional report. *Right panel*: investigations initiated by report from any nonmedical reporter.

**FIG. 2.**
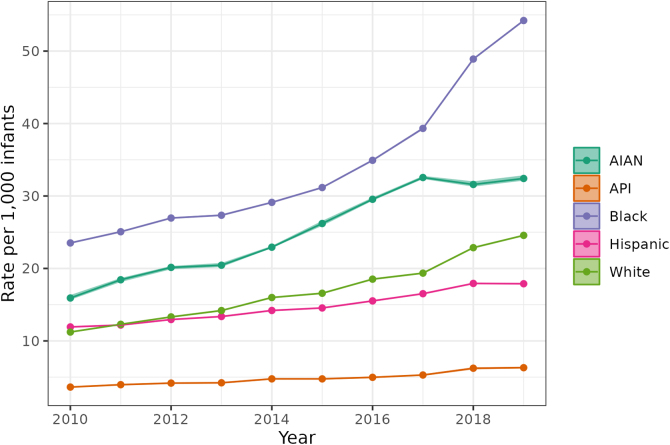
U.S. child welfare investigations of infants (age <1 year) initiated following a medical professional report, 2010–2019 by child race/ethnicity. *Intervals* indicate uncertainty from missing race/ethnicity data.

We provide two sets of descriptive estimates of child welfare investigation rates. The first (Figs. 1 and 2) evaluates patterns in maltreatment investigations between 2010 and 2019 for all 50 U.S. States and D.C. We examine counts and population-adjusted rates of investigations for infants (0 through 1 year old) by: source of initial child welfare report, primary type of alleged maltreatment, and infant race/ethnicity.

Infant race/ethnicity is treated as a single-value categorical variable, as identified by the investigating caseworker. NCANDS provides nonmutually exclusive categorical indicators for child race/ethnicity in the following groups: American Indian or Alaska Native; Asian; Black or African American; Native Hawaiian or other Pacific Islander; White; unable to determine; Hispanic or Latino ethnicity. To harmonize these data with available age-specific population estimates, we reduce these seven race/ethnicity variables to a single categorical variable that takes the following mutually exclusive values: American Indian or Alaska Native (AIAN) alone or in combination, Black alone or in combination (non-AIAN), Asian or Pacific Islander alone or in combination (non-AIAN, non-Black), Hispanic (White alone), and White.

NCANDS documents sources of reports for all investigations with a 15-categorical variable. The variable identifies “medical personnel” as one report source. We treat all cases of missing values on source of report as originating from nonmedical sources. In line with prior research, we assume mandated reporters of child maltreatment operating in official capacities are more likely to be identified affirmatively in administrative records.^34^ We compare child welfare investigations originating from medical professional reports to all other sources of investigated reports.

NCANDS also records variables documenting types of child maltreatment involved in cases. We construct binary indicators for alleged maltreatment types: neglect, physical abuse, sexual abuse, and other. The definition of neglect includes cases coded as medical neglect. The U.S. Children's Bureau advises states to code substance exposure as neglect for data collection purposes. We then compare maltreatment types in investigations stemming from medical professional reports to those from all other sources over time for all U.S. states and D.C.

States are required to count cases of infant or prenatal substance exposure (IPSE) reported to child welfare agencies. The U.S. Children's Bureau provides annual counts of IPSE cases using NCANDS by identifying children (1) under 1 year of age at time of report; (2) reported to child welfare agencies by medical professionals; and (3) whose report involves an allegation of substance use (a binary measure derived from four categorical variables: drug abuse—child, drug abuse—caregiver, alcohol abuse—child, alcohol abuse—caregiver).^1^ We rely on this operational definition in our analysis to compute state, year, and race-specific IPSE case counts and rates for all states with valid data between 2010 and 2019. We also compute similar case counts and rates for all investigations of infants and by report source.

Despite a legal requirement to document IPSE-involved investigations, there are measurement limitations with IPSE data in NCANDS. Because each state compiles and submits data individually to the Children's Bureau, data quality varies across states and within states over time. In 2019, the Children's Bureau identified four states that failed to submit IPSE data. Our analysis finds that only 25 states adequately recorded IPSE data in NCANDS in 2019, and that only 11 states have adequately recorded IPSE data in NCANDS over the full 2010–2019 period. We count a state as having invalid IPSE data for a given year if it meets the following criteria: (1) state is identified by U.S. Children's Bureau as having invalid data; (2) state has >20% of cases missing data on the IPSE index described previously; and (3) fewer than 1 in 10,000 infants in the state are identified as IPSE involved, a conservative threshold given prior prevalence estimates.^36,41,43^

Many states with valid IPSE data still have substantial proportions of cases missing data on variables used to identify IPSE cases. We construct multiple imputed datasets using chained equations to describe how missing data impacts uncertainty in estimates of IPSE investigation rates.^46^ Missing IPSE data for states with generally high-quality data are modeled using logistic regression, missing race/ethnicity variables are modeled using multinomial regression, and missing age data are modeled using partial mean matching. All intervals reported here reflect complete postimputation uncertainty.

We couple all counts of unique infants with child welfare investigations with population data from the National Cancer Institute's Surveillance, Epidemiology, and End Results program. These data provided bridged-race annual county-level population estimates for single-year age groups derived from U.S. Census small area estimates. We aggregate these data to the state level to provide annual population totals for each of five racial/ethnic groups between age 0 and 1, then compute infant investigation event rates as events per 1000 infant population for each group. We use these estimated event rates to describe variation in IPSE child welfare investigations nationally, across states, and within states over time. We also estimate state-level rate ratios to evaluate magnitude of racial/ethnic differences in investigations.

## Results

Between 2010 and 2019, there were 2,835,139 [postimputation uncertainty interval: 2,833,872–2,836,475] infants who were subjects of child welfare investigations in the United States. Of these infants, 25.8% (*n*=731,595 [731,491–731,705]) were investigated following a report from a medical professional. Figure 1 provides trends in numbers of infant maltreatment investigations for all states and D.C. following reports by reporter type and by alleged maltreatment type(s).

In 2010, there were 51,868 [51,812–51,919] infants investigated following a medical professional's report. In 2019, there were 102,636 [102,589–102,682] such investigations, nearly double that in 2010. In 2010, reports by medical professionals initiated ∼21% of all child welfare investigations of infants. This rate increased to 34% in 2019. Investigations from all other reporter categories remained relatively stable during this period.

The overwhelming majority of maltreatment reports filed by medical professionals (and other reporters) center on concerns of neglect. In 2019, 72% of investigations following a medical professional's report contained an allegation of neglect, and 29% contained an allegation of physical abuse.

Figure 2 provides national trends in infants investigated by child welfare following a medical professional's report. In each year between 2010 and 2019, AIAN infants and Black infants were more likely to be investigated following a medical professional's report than were White, Hispanic, or Asian/Pacific Islander infants. Rates of AIAN investigations grew from 15.9 per 1000 infants in 2010 to 32.4 per 1000 infants in 2019, growth of 203%. Black infants were investigated at a rate of 23.5 per 1000 in 2010 and 54.2 per 1000 in 2019, growth of 231%. White children were investigated at a rate of 11.2 per 1000 in 2010 and 24.6 per 1000 in 2019, growth of 220%. Relative increases from 2010 to 2019 were similar across racial/ethnic groups, but there were differences in absolute increases, with an additional 1.3% of White, 1.7% of Indigenous, and 3.1% of Black infants investigated in 2019 as compared with 2010.

**FIG. 3.**
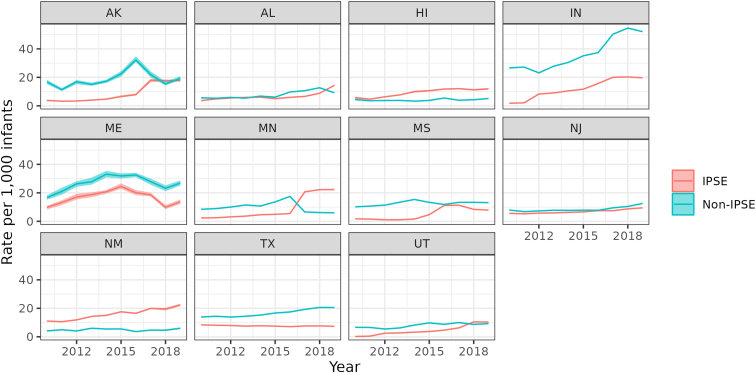
Child welfare investigations of infants (age less than 1 year) reported by medical professionals 2010–2019 with and without IPSE in select states with high-quality data. IPSE, infant or prenatal substance exposure.

Figure 3 provides trends in investigations of infants following reports by medical professionals for both IPSE and non-IPSE cases for the 11 states with valid IPSE data for the full study period. In nine of these states, rates of IPSE investigations increased between 2010 and 2019. Across these 11 states, IPSE cases comprised 32% of all investigations of infants following a medical professional report in 2010. By 2019, this proportion had risen to 38%. In some states, the proportion was greater. In Minnesota in 2019, 79% investigations following a medical professional's report involved IPSE.

**FIG. 4.**
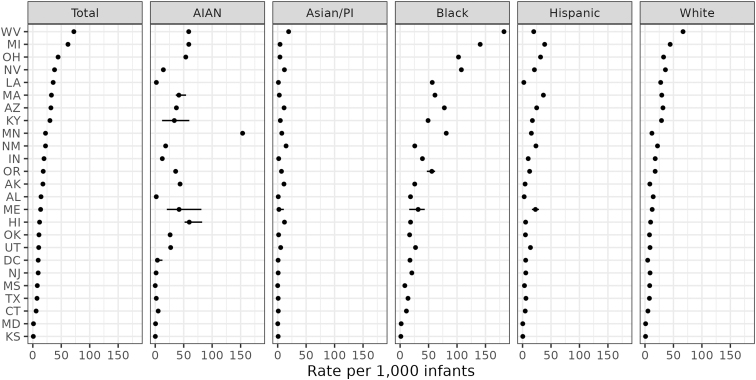
Child welfare investigations of infants (age <1 year) reported by medical professionals 2010–2019 with IPSE by race/ethnicity in select states with high-quality data, 2019. *Bars* indicate uncertainty intervals.

IPSE data quality has improved in NCANDS over time. Whereas only 11 states had valid data over the full 2010–2019 period, 25 had valid data for 2019. Race/ethnicity-specific rates of investigations for IPSE are given in Figure 4 for these 25 states in 2019. For this set of states, we estimate that 22 per 1000 infants were investigated for IPSE following a medical professional's report to child welfare. In 2019, 29 per 1000 AIAN infants were investigated, 5 per 1000 API infants were investigated, 44 per 1000 Black infants were investigated, 11 per 1000 Hispanic infants were investigated, and 20 per 1000 White infants were investigated.

IPSE investigation rates vary widely across states and racial/ethnic groups. For all infants, rates range from a maximum of 72 IPSE investigations per 1000 infants in West Virginia to about 1 per 1000 in Kansas and Maryland. More than 1 in 10 Black infants in West Virginia, Michigan, Ohio, and Nevada were investigated for IPSE in 2019. AIAN children also face exceptionally high levels of investigation for IPSE in some states. In Minnesota in 2019, for example, 150 per 1000 AIAN infants were investigated following an IPSE report to child welfare. The highest 2019 IPSE investigation rate for API infants was 19 per 1000 in West Virginia. The highest for Hispanic infants was 39 per 1000 in Michigan, and the highest for White infants was 67 per 1000 in West Virginia.

**FIG. 5.**
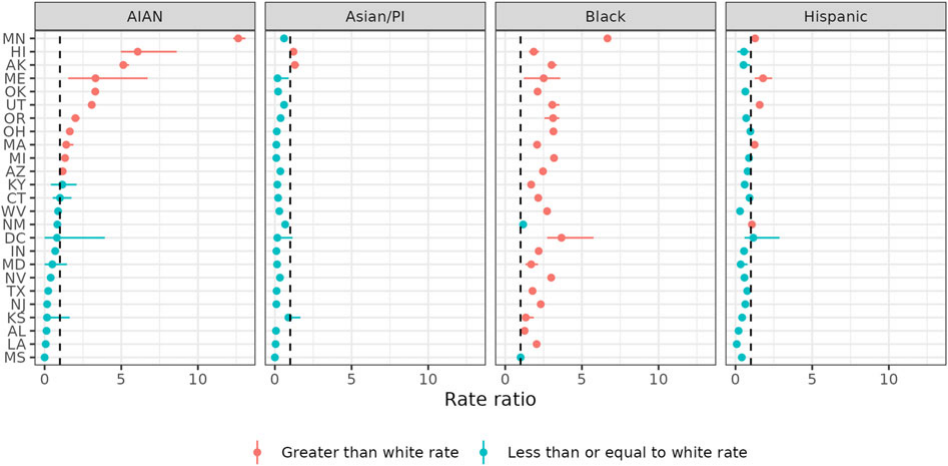
Inequity in child welfare investigations of infants (age less than 1 year) reported by medical professionals 2010–2019 with IPSE by race/ethnicity in select states with high-quality data, 2019. Rate ratio relative to White. *Dashed line* indicates equality. *Bars* indicate uncertainty intervals. *Color* indicates statistical significance.

Figure 5 provides 2019 IPSE data as rate ratios for AIAN, API, Black, and Hispanic relative to White infants in each state. This figure shows likelihood that an infant of color experienced an IPSE investigation in 2019 in each state relative to a White infant. Color shows whether the postimputation rate ratio is greater than 1, indicating statistically significant inequity. Among these 25 states, Minnesota exhibits the highest levels of inequity for both AIAN and Black infants. AIAN infants are investigated for IPSE 13 times more frequently than White infants in the state. Black infants in Minnesota are investigated for IPSE seven times more frequently than White infants. AIAN infants are more likely to be investigated for IPSE than White infants in 11 of these 25 states. Black infants are more likely to be investigated for IPSE than White infants in 23 of these 25 states. Compared with White infants, Hispanic infants are more likely to be investigated for IPSE in five states and API infants in two states.

## Discussion

This study provides the first multistate prevalence estimates of infants investigated for IPSE referred by medical professionals and situates IPSE reporting in the broader context of increasing child welfare investigations of infants following reports by medical professionals. Specifically, we found that medical professional reports have become an increasingly common source of child maltreatment investigations of infants over time, whereas rates of reporting for other mandated reporters remained relatively stable over this period. This contrast suggests shifts in rules and practices of infant maltreatment surveillance in health care have resulted in an increase in the number of infants entering the child welfare system. This notable increase may be related, in part, to the 2016 federal Comprehensive Addiction and Recovery Act, as states and hospitals updated reporting policies to comply with new regulations, something to explore in future research.

We also found that racial inequities in child welfare investigations of infants persist, although the magnitude of inequity varies by state. Per capita rates of investigations of Black infants following medical professionals' reports increased ∼230% between 2010 and 2019, a relative increase similar to relative increases in investigations of Indigenous and White infants. Of note, though, the absolute increase in investigations of Black infants was >1.5% greater for Black than White and Indigenous infants. This increase means that, in 2019, >5% of Black infants in the United States were the subject of a child welfare investigation that originated from a medical professional's report. As child welfare system contact may harm rather than improve health, racial inequities in investigations may contribute to broader health inequities for Black and Indigenous birthing people and infants.^17,18,22–25,27–31^

Following prior research, we identify reports classified as IPSE to be major contributors to overall rates of child welfare investigations of infants. In 2019, child welfare agencies investigated ∼2.2% of infants for IPSE, greater than prior estimates from studies conducted earlier in the 2000s and in smaller geographic areas.^36,43,44^ We also found rates of IPSE investigations varied significantly across states (7.2% of infants investigated in West Virginia vs. 0.1% in Maryland), suggesting that estimates of IPSE investigations based on data from single geographic areas should not be assumed to generalize to the rest of the United States. The study also confirmed that significant racial inequities in IPSE investigations persist, with more than two times as many Black than White infants and one and a half times as many AIAN than White infants investigated for IPSE.

All estimates should be interpreted as conservative lower bounds on actual rates of medical professionals' reports of infants to child welfare. Federal data systems do not record detailed information on cases reported to but not investigated by child welfare agencies. Overall child maltreatment reporting data indicate that 40–50% of reports made overall are not investigated,^1^ indicating that rates of medical reporting to child welfare agencies are almost certainly higher than rates of investigation, which are what NCANDS data capture. In addition, there is considerable heterogeneity in data collection practices on IPSE variables across states. Although the U.S. Children's Bureau reports five states as lacking IPSE data in 2019,^1^ we identified 25 states with invalid data in 2019. High volumes of missing data and implausibly low prevalence are a function of variation in policies and practices in recording and reporting relevant measures across states and within states over time.^48^

This study cannot disentangle reasons for changes in rates of investigations over time. We do note, though, that increases in opioid use in the United States created political pressure for widespread policy and practice changes in surveillance, reporting, and investigations at federal, state, local, and institutional levels. Research suggests that these state policy changes have contributed to increases in reporting and investigations.^41^ As such, observed changes over time should not be attributed solely to changes in substance use. Because prior research suggests that these changes in reporting have disproportionately impacted infants of color and their families ^41,49,50^ and may harm the children they are intended to help,^17,26,27,51^ policymakers should pause any pending expansions of mandated reporting and consider repeals of prior expansions. Further research should seek to explain increases in reporting by considering the possible contributions of (1) actual changes in substance use among birthing people; (2) federal and state laws; (3) reporting practices by medical professionals; and (4) child welfare agency data collection.

Medical professionals concerned about increases in reporting can review recent research on the impact of child welfare interventions, particularly in relation to parental substance use^25,36,52–54^ and reflect on the possible consequences of overreporting to child welfare, as well as what their roles might be in reducing overreporting.^55^ Furthermore, to inform an understanding of what alternative policies and supports might look like, health professionals and policy makers should connect with community-based organizations led by people directly affected by these policies—such as JMACForFamilies,^55^ Rise,^56^ and Movement for Family Power^57^– to identify alternatives policies and supports that are more relevant and helpful to children and families.

## Conclusion

Child welfare investigations of infants resulting from reports by medical professionals have increased notably since 2010. Medical professional reports are a significant contributor to the stark racial inequities in these child welfare investigations both in general and specifically related to substance use.

## Abbreviations Used

AIAN: American Indian/Alaska Native
CARA: Comprehensive Addiction and Recovery Act of 2016
IPSE: Infants related to prenatal substance exposure
NCANDS: National Child Abuse and Neglect Data System
